# Stability analysis of rainfall-induced landslide considering air resistance delay effect and lateral seepage

**DOI:** 10.1038/s41598-024-59121-4

**Published:** 2024-04-10

**Authors:** Li Li, Hanjie Lin, Yue Qiang, Yi Zhang, Siyu Liang, Shengchao Hu, Xinlong Xu, Bo Ni

**Affiliations:** Department of Civil Engineering, Chongqing Three Gorges University, Wanzhou, Chongqing, 404100 China

**Keywords:** Green-Ampt infiltration model, Slope stability, Air resistance delay, Lateral seepage, Natural hazards, Civil engineering

## Abstract

Accumulation landslides are prone to occur during the continuous infiltration of heavy rainfall, which seriously threatens the lives and property safety of local residents. In this paper, based on the Green-Ampt (GA) infiltration model, a new slope rainfall infiltration function is derived by combining the effect of air resistance and lateral seepage of saturated zone. Considering that when the soil layer continues to infiltrate after the saturation zone is formed, the air involvement cannot be discharged in time, which delays the infiltration process. Therefore, the influence of air resistance factor in soil pores is added. According to the infiltration characteristics of finite long slope, the lateral seepage of saturated zone is introduced, which makes up for the deficiency that GA model is only applicable to infinite long slope. Finally, based on the seepage characteristics of the previous analysis, the overall shear strength criterion is used to evaluate the stability of the slope. The results show that the safety factor decreases slowly with the increase of size and is inversely correlated with the slope angle and initial moisture content. The time of infiltration at the same depth increases with the increase of size and slope angle, and is inversely correlated with the initial moisture content, but is less affected by rainfall intensity. By comparing with the results of experimental data and other methods, the results of the proposed method are more consistent with the experimental results than other methods.

## Introduction

Heavy rainfall infiltration often leads to slope instability and poses a serious threat to the lives and property of local residents. At present, the stability evaluation of rainfall-induced landslides is mainly divided into two steps. The first is to select the appropriate rainfall infiltration model to analyze its infiltration mechanism and hydraulic effect. Then, based on this, the safety factor of the landslide is calculated after the stress analysis. In addition, satisfactory analysis (including artificial intelligence, etc.) of risk assessment constituted an active area of research in the field of slope stability and landslides^1,2^. And combining statistical analysis and physical modeling for risk assessment can better understand the conditions of landslide and slope stability in different environments.

Therefore, it is very important to construct an accurate rainfall infiltration model to predict the water movement inside the soil during rainfall for analyzing slope stability. At present, such methods are mainly divided into theoretical infiltration model and approximate infiltration model^3,4^. Among them, the theoretical infiltration model is usually based on the assumption of continuous media to propose partial differential equations (such as Richards equation) to describe the infiltration process of water in soil, and solved by integral transformation or other numerical methods^5–8^. The approximate infiltration model is usually based on the principle of water balance and Darcy's law to simplify the calculation process. Due to the use of fewer parameters to obtain more accurate results, such methods have become popular^4,9^. Green-Ampt rainfall infiltration model is a typical example of this method.

The Green-Ampt rainfall infiltration model was originally proposed to be applied to the horizontal soil in the agricultural field^10^. After Chen et al. expanded the model to the inclined surface, it is now receiving more and more attention in the study of rainfall-induced landslides^11–14^. The model has clear physical meaning and is easy to solve. It has great potential in analyzing the infiltration mechanism of shallow rainfall landslide. Over the years, many scholars have made a lot of supplements to the scope of use of the model, the characteristics of mechanical parameters and influencing factors^15–18^. For example, Almedeij and Esen proposed an improved GA model based on stable rainfall intensity conditions based on Mein and Larson's classical extension model^19,20^. Zhang et al. calculated the pore water pressure distribution similar to the Richards equation based on the non-uniform distribution of initial water content^21^. Gavin and Xue proposed that the matric suction of unsaturated soil changes during rainfall infiltration, and analyzed a new model of matric suction variation^22^. In addition, some new ideas have been put forward in recent years. Li et al. believed that the rhizomes of plants have a certain influence on the stability of the slope after infiltration^23^. Xu et al. applied the improved GA infiltration model to the stability analysis of three-dimensional slope^24^. Meng and Yang combined BP neural network fitting parameters to partition the wet zone^25^. Although these scholars have done many researches on the GA model, the theory still has great potential for development.

In summary, considering that there are few studies on the model in some specific soils (such as sandy soil with large porosity), and for the next step, the model is applied to the finite grid of GIS (Geographic Information System) for regional numerical simulation. This article will make improvements in the following two directions: (1) For the large porosity under certain conditions, there will be a lag of landslide relative to rainfall^26^. When the infiltration saturation zone is formed, the air in the soil pore cannot be eliminated in time and is compressed, the infiltration process may be delayed. (2) When using the GA model, the research object is regarded as an infinite slope^27,28^. In fact, the slope is a finite length, especially in the rainfall landslide test cannot ignore its size effect.

This paper first considers the influence of air resistance on infiltration rate. Then, a new GA infiltration model suitable for finite long slope is obtained by combining the lateral seepage effect of saturated zone. Based on the previous analysis of infiltration characteristics, the overall strength criterion is used to analyze the stability of the slope. Finally, the reliability is verified by comparing with other methods and test results.

## Model construction

### Improved Green-Ampt infiltration model

**Figure 1:**
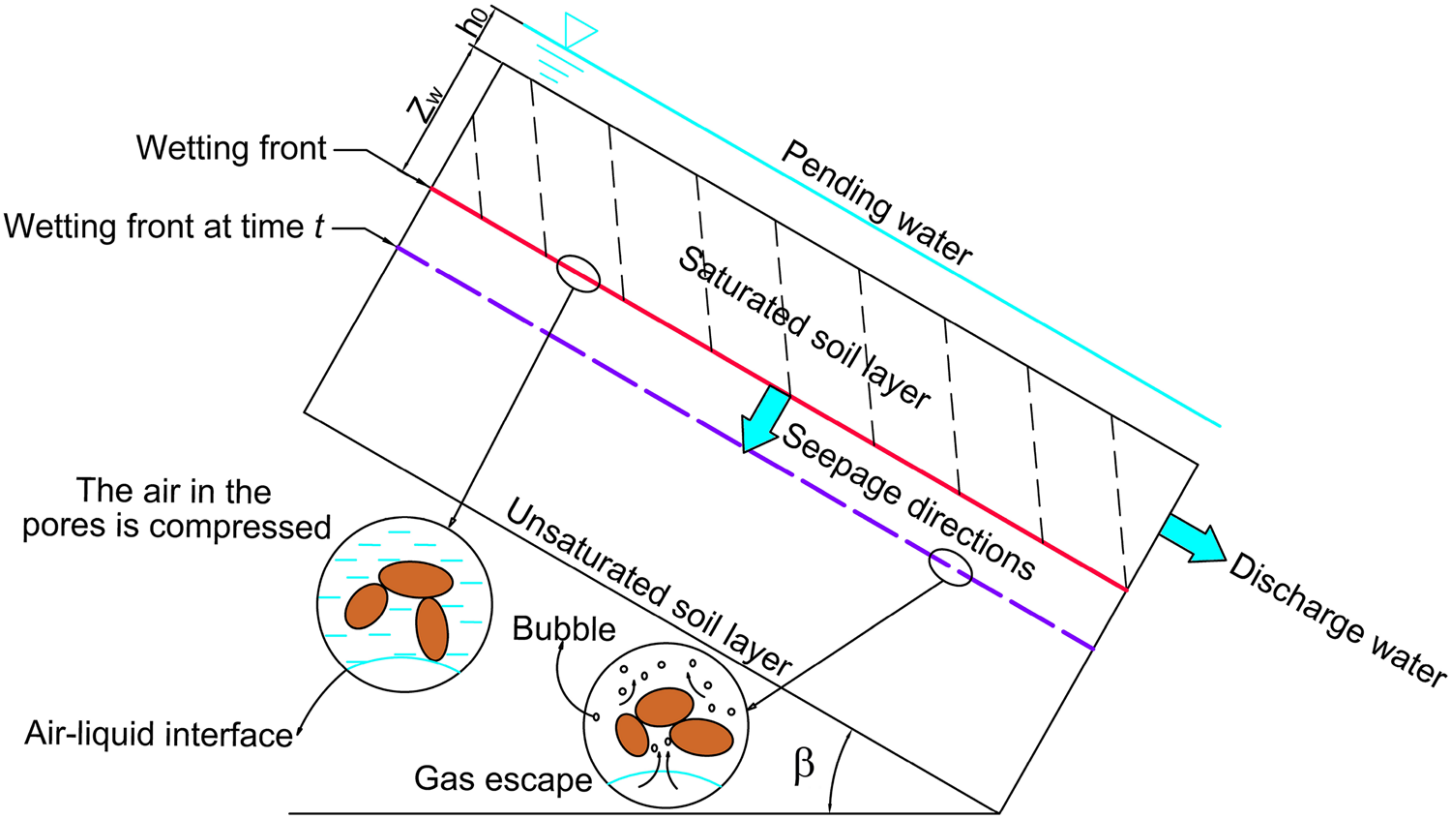
Improved Green-Ampt infiltration model.

The analysis of slope rainfall infiltration mechanism is the basis for studying slope stability. In the early stage of rainfall, the infiltration rate was controlled by rainfall intensity, but after the slope began to accumulate water, the infiltration rate was mainly affected by the combined action of water head and matrix suction. Therefore, when the slope begins to accumulate water, because the air in the soil layer cannot be removed from the slope in time, the air in the soil layer is compressed to a certain value and escapes with the wetting front moves down. The repetition of this process will delay the rainfall infiltration time. In addition, part of the saturated zone water is discharged along the slope inclination direction under the control of the slope geometry and hydraulic gradient. The above two phenomena are the basis for us to improve the Green-Ampt infiltration model. The model As shown in Fig. 1.

Before analyzing the rainfall infiltration process of the slope, the following assumptions are made to the model:

1. The upper soil layer is homogeneous soil, and the lower part is impermeable bedrock.
2. Without considering the effect of groundwater, assuming that the soil moisture content is uniform, the matric suction head is a fixed value.
3. The rainfall intensity is greater than the saturated permeability coefficient of the soil and the rainfall direction is vertical downward.

After the formation of the wetting front (the interface between the unsaturated zone and the saturated zone), the Green-Ampt model suitable for the slope was proposed to represent the infiltration rate of this process^11^.

1 $$i={K}_{s}\frac{{z}_{w}\mathit\,{cos}\,\beta +{\psi }_{f}+{h}_{0}}{{z}_{w}}.$$

In this paper, an improved Green-Ampt infiltration model suitable for slopes is proposed after considering the effect of air resistance:

2 $$i={K}_{s}\frac{{z}_{w}\mathit\,{cos}\,\beta +{\psi }_{f}+{h}_{0}-{h}_{af}}{{z}_{w}}.$$

In the formula: *i* represents infiltration rate; *K*_*s*_ represents the saturated permeability coefficient of soil; *z*_*w*_ denotes the vertical depth to the slope surface; *ψ*_*f*_ is the matric suction; *β* is the slope angle; *h*_*0*_ represents the water head of the slope area and *h*_*af*_ represents the average gas resistance.

As previously analyzed, gas resistance is not continuous. In view of this, Wang et al. divided the change of air resistance in the infiltration process into two stages: air compression and air escape^29^. The air escape value *H*_*b*_ and the air closure value *H*_*c*_ are used to represent the pressure head in the pores. *H*_*b*_ and *H*_*c*_ are expressed as follows:

3 $${H}_{b}={h}_{0}+{z}_{w}\mathit\,{cos}\,\beta +{h}_{ab},$$

4 $${H}_{c}={h}_{0}+{z}_{w}\mathit\,{cos}\,\beta +{h}_{wb},$$

where *h*_*ab*_ represents the air-bubbling capillary pressure value; *h*_*wb*_ represents the water-bubbling value.

herefore, the pore gas pressure *h*_*af*_ considering air entrapment can be expressed as:

5 $${h}_{af}=\frac{{H}_{b}+{H}_{c}}{2}={h}_{0}+{z}_{w}\mathit\,{cos}\,\beta +\frac{{h}_{ab}+{h}_{wb}}{2}.$$

According to the previous definition, the critical time for the formation of the wetting front during infiltration is *t*_*p*_. Referring to the analysis and data fitting by Zhang et al. the critical wetting front depth *z*_*p*_ can be expressed as Ref.^26^:

6 $${z}_{p}=\frac{{K}_{s}\left({\psi }_{f}+{h}_{0}\right)}{{e}^{-({e}^{2q}-{K}_{s})}\mathit\,{cos}\,\beta }.$$

Similarly, *z*_*p*_ can be seen as the critical depth of infiltration when the ponding occurs, then the rainfall intensity is equal to the infiltration rate *i*:

7 $$q\mathit\,{cos}\,\beta ={K}_{s}\frac{{z}_{p}\mathit\,{cos}\,\beta +{\psi }_{f}+{h}_{0}-{h}_{af}}{{z}_{p}}.$$

Therefore, when the wetting front depth is *z*_*p*_, the relationship between cumulative infiltration *I*_*p*_ and soil water content is as follows:

8 $${I}_{p}={\int }_{0}^{{z}_{p}}[{\theta }_{s}-{\theta }_{i}]dz=\left({\theta }_{s}-{\theta }_{i}\right){z}_{p}.$$

In the formula, *θ*_*s*_ represents the saturated volumetric water content of the soil, and *θ*_*i*_ represents the natural volumetric water content of the soil. Then the critical time *t*_*p*_ can be expressed as:

9 $${t}_{p}=\frac{{I}_{p}}{q\mathit\,{cos}\,\beta }=\frac{\left({\theta }_{s}-{\theta }_{i}\right){z}_{p}}{q\mathit\,{cos}\,\beta }.$$

When the saturated zone is formed, part of the water flows laterally along the slope inclination during the wetting front moving downward. Because the lateral seepage of saturated zone water will reduce depth of the wetting front, the cumulative infiltration of Eq. (8) combined with Darcy's law can be obtained:

10 $${K}_{s}\mathit{sin}\beta {z}_{f}dt=\left({\theta }_{s}-{\theta }_{i}\right)Ld{z}_{f},$$

where *L* represents the length of the slope surface.

The derivative of the cumulative infiltration *I*_*p*_ to time *t* is equal to the infiltration rate *i*, so the decrease rate of the wetting front after considering the lateral seepage of the saturated zone can be obtained as follows:

11 $$\left(\frac{d{z}_{f}}{dt}\right)={K}_{s}\frac{{z}_{f}\mathit\,{sin}\,\beta }{\left({\theta }_{s}-{\theta }_{i}\right)L}.$$

Finally, referring to the formula of wetting front with time obtained by Zhang et al., the variation of wetting front depth with time is obtained after considering the gas resistance effect and lateral seepage^21^. Among them, in view of the fact that the soil layer thickness of rainfall infiltration is often shallow, the influence of lateral seepage is simplified to the ratio of depth to time.

12 $$={t}_{p}+\frac{\Delta \theta \left({z}_{w}-{z}_{p}\right)}{{K}_{s}\mathit\,{cos}\,\beta }-\frac{\Delta \theta \left({\psi }_{f}-{h}_{af}\right)}{{K}_{s}{\mathit\,{cos}}^{2}\,\beta }\mathit{log}10\left(\frac{{\psi }_{f}+{z}_{w}\mathit\,{cos}\,\beta }{{\psi }_{f}+{z}_{p}\mathit\,{cos}\,\beta }\right)-\frac{H}{\mathit\,{cos}\,\beta }\frac{L\Delta \theta }{{K}_{s}{z}_{w}\mathit\,{sin}\,\beta }.$$

It can be seen from formula (12) that the variation of wetting front with time is affected by gas resistance, which is helpful to optimize the infiltration research of large porosity soil such as sand and laterite. After considering the lateral seepage, the infiltration time is related to the slope size, which is conducive to the future application in the finite element numerical simulation.

### Slope stability analysis

At present, some studies calculated the slope stability coefficient based on the shear strength of unsaturated soil, only considering the increase of soil weight by rainfall^16^. Obviously, other stress changes caused by rainfall are also necessary to analyze. In this paper, the stability of slope is analyzed with bedrock as sliding surface. When the wetting front is close to the bedrock, the air resistance formed by the compression of the wetting front should be added to the force analysis^26^. Due to the shallow thickness of the slope soil layer, the air compression resistance is added to the whole stress analysis process. In addition, for a finite length slope, the seepage force caused by the lateral seepage of the saturated zone has an effect on the slope stability^30^. Finally, on this basis, the overall shear strength is used to analyze the stability of the slope.

#### Shear strength of soil block

The shear strength criterion of soil is the basis of the limit equilibrium method, so the appropriate strength criterion should be selected before the stability analysis of the slope.

For unsaturated soils under natural conditions, the widely used shear strength criterion is the Mohr–Coulomb expansion criterion proposed by Fredlund^31^. The theory reflects the strength of unsaturated soil through independent double stress states. As shown below:

13 $$\tau ={c}^{\prime}+\left(\sigma -{u}_{a}\right)\mathit\,{tan}\,{\varphi}^{\prime}+\left({u}_{a}-{u}_{w}\right)\mathit\,{tan}\,{\varphi }^{b}$$

where σ denotes the total normal stress; *c'* and *j'* represent the effective cohesion and effective internal friction angle of soil; *u*_*a*_ and *u*_*w*_ represent pore air pressure and pore water pressure of soil; *u*_*a*_*-u*_*w*_ represents matrix suction; *φ*^*b*^ is the friction angle used to indicate the increase of matric suction.

Since normal stress and matric suction are two independent variables, matric suction can be regarded as a part of cohesion. The apparent cohesion is then defined as the sum of matric suction and effective cohesion as follows:

14 $$\tau ={c}_{\psi }^{*}+\left(\sigma -{u}_{a}\right)\mathit\,{tan}\,{\varphi}^{\prime}$$

15 $${c}_{\psi }^{*}={c}^{\prime}+\left({u}_{a}-{u}_{w}\right)\mathit\,{tan}\,{\varphi }^{b}$$

**Figure 2:**
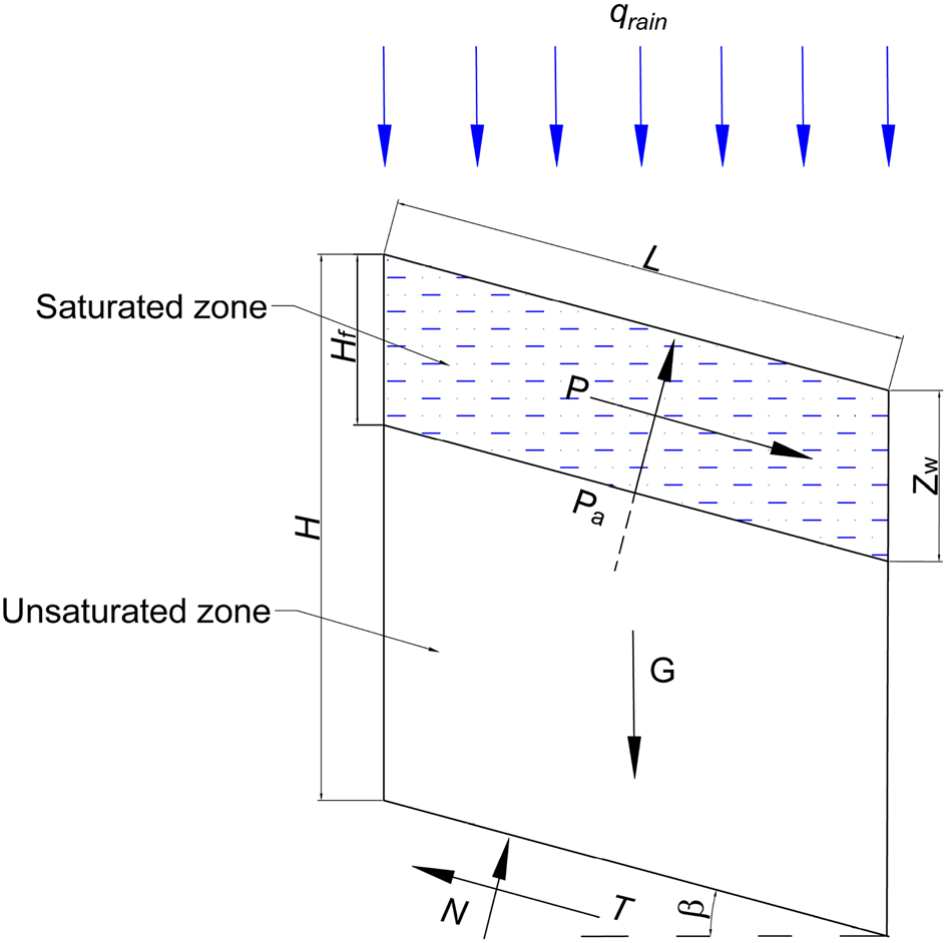
The stress indication of soil block.

During the actual rainfall infiltration process, the moisture content in the slope is not uniform. The soil layer above the wetting front first reaches saturation, while the soil layer below the wetting front is unsaturated. Montrasio and Valentino proposed a simplified shear strength calculation model based on laboratory tests^32^. As shown in Fig. 2, the bar with a height of *H* and a wetting front depth of *H*_*f*_ is simplified to a new uniform water content calculation. The effective internal friction angle of the new method is unchanged, while the relationship between the apparent cohesion and the original method is as follows:

16 $${c}_{\psi }={c}^{\prime}+\left({u}_{a}+{u}_{w}\right)\mathit\,{tan}\,{\varphi }^{b}{\left(1-\eta \right)}^{3.4}$$

In the formula, *η* is *H*_*f*_*/H*, *H*_*f*_ denotes the vertical height of saturated soil, expressed as *H*_*f*_ = *Z*_*w*_*/cosβ*, 3.4 is the test parameter^32^.

Then, the overall shear strength of the soil block is expressed as:

17 $$\tau =\left(\sigma -{u}_{a}\right)\mathit{tan}\,{\varphi}^{\prime}+{c}_{\psi }$$

#### Stability coefficient calculation

Based on the above analysis of the stress of the soil block. *G* is the vertical downward soil gravity; *T* is the anti-sliding force parallel to the sliding surface and the support force *N* of the sliding surface. In addition, *P* and *P*_*a*_ are shown in Eqs. (19) and (20). Stress diagram as shown in Fig. 2:

Therefore, the gravity of the soil block can be expressed as:

18 $$G={\gamma }_{s}{z}_{w}L\mathit\,{cos}\,\beta +\gamma \left(H-{z}_{w}\right)L\mathit\,{cos}\,\beta$$

Among them, *L* represents the length along the slope direction, *H* is the thickness of the soil layer, *γ*_*s*_ and *γ* represent the saturated unit weight and natural unit weight of the soil.

Considering the lateral seepage of the saturated zone, the lateral seepage force *P* parallel to the sliding surface is expressed as:

19 $$P={\gamma }_{w}{z}_{w}L\mathit\,{sin}\,\beta\mathit\,{cos}\,\beta$$

The gas resistance *P*_*a*_ produced when the wetting front moves down is as follows:

20 $${P}_{a}={\gamma }_{w}{h}_{af}{\mathit{cos}}^{2}\beta +{u}_{a}$$

Finally, the landslide stability coefficient *F*_*s*_ is defined as the ratio of anti-sliding force to sliding force. Based on the previous stress analysis, it is obtained that:

21 $${F}_{s}=\frac{\left(G-\mathit\,{cos}\,\beta {P}_{a}\right)\mathit\,{cos}\,\beta \mathit\,{tan}\,{\varphi}^{\prime}+L{c}_{\psi }}{G\mathit\,{sin}\,\beta +P}$$

When *F*_*s*_ is greater than 1, the slope is regarded as stable; when Fs is less than 1, the slope is considered unstable. On the basis of predecessors, this formula adds lateral seepage force and gas resistance, which is more suitable for the actual situation of finite length slope.

## Result analysis and verification

**Figure 3:**
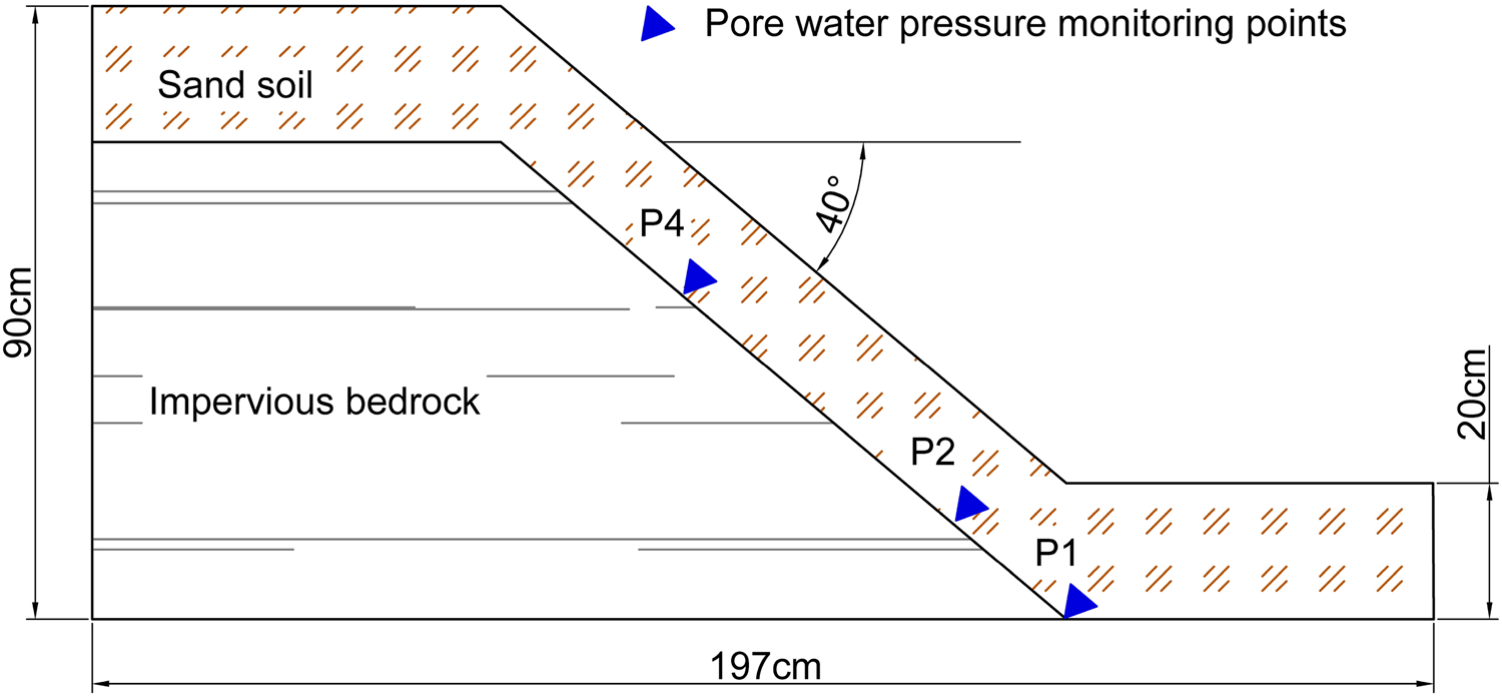
Landslide rainfall model test diagram (Orense et al. 2004).

Orense and Shimoma carried out several indoor model experiments to study the mechanism of rainfall-induced landslides^33,34^. This paper takes Orense's indoor model test as an example to verify the reliability of the proposed method. The lower part of the model is impervious bedrock and the upper cover layer is 20 cm sand. As shown in Fig. 3:

The soil physical parameters are as follows: *K*_*s*_ = 0.018mm/s, *θ*_*s*_ = 0.45, *θ*_*i*_ = 0.1, *c* = 0kPa, ${\varphi}^{\prime}$=36°. In addition, the rainfall intensity q_rian_ = 262mm/h, the slope angle is *β* = 40°and three pore water pressure monitoring points are set along the interface between sand and bedrock.

**Figure 4:**
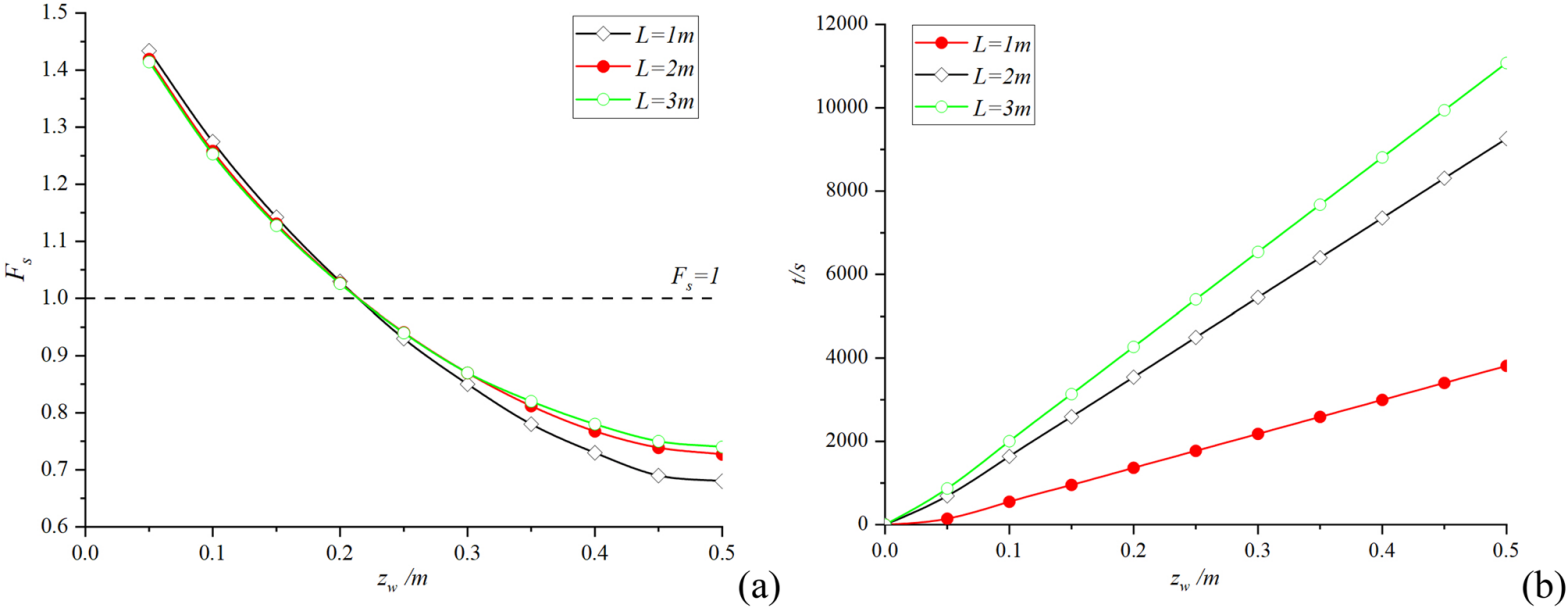
Size effect analysis: (**a**) The change of safety factor with slope length; (**b**) The change of infiltration time with slope length.

During the experiment, when the rainfall continued to about 1800s, cracks appeared at the top of the slope; when it continues to about 3500 s, the slope has a large displacement. Based on the above test parameters, in order to verify the reliability of the theoretical improvement in this paper, the following results are obtained by using the proposed method when assumed the soil layer thickness is 0.5 m (this paper uses Matlab2019a mathematical software to calculate). The influence of slope length *L* on safety factor *F*_*s*_ and infiltration time *t* during the whole infiltration process is shown in Fig. 4:

It can be seen from Fig. 4a that the larger the size *L*, the smaller the safety factor *F*_*s*_ in the initial stage of rainfall, but with the deepening of infiltration depth, the safety factor *F*_*s*_ of large slope decreases more slowly. Combined with the theoretical derivation analysis in Section "Slope stability analysis", the initial stage has a lower safety factor *F*_*s*_ due to the greater gravity *G* of the soil block. However, the accommodation capacity of large-scale slopes is stronger, so the safety factor *F*_*s*_ of large-scale slopes decreases more slowly during continuous rainfall. It can be seen from the similar analysis Fig. 4b that the infiltration time *t* increases with the increase of the slope size *L*, which conforms to the theoretical derivation and the actual situation.

**Figure 5:**
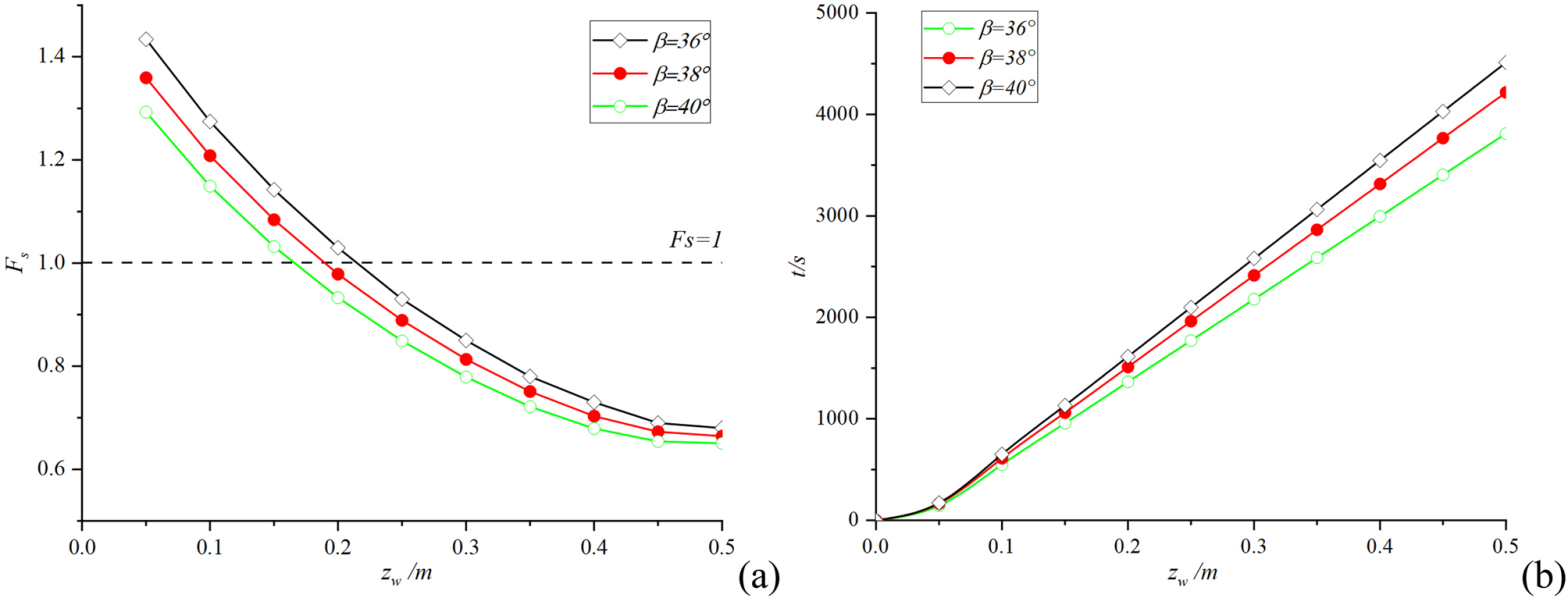
Slope angle effect analysis: (**a**) The change of safety factor with slope angle; (**b**) The change of infiltration time with slope angle.

The influence of slope angle *β* on infiltration time *t* in the whole infiltration process is shown in Fig. 5:

By analyzing Fig. 5a, the safety factor Fs reaches the critical value 1 faster with the increase of slope angle b. It can be seen from Fig. 5b that the infiltration time t increases with the increase of slope angle b. Combined with the previous infiltration principle analysis, when the slope angle b increases, the water flow on the slope surface accelerates, so the water head h0 decreases. In addition, as shown in Eq. (10), the influence of lateral seepage on infiltration rate increases with the increase of slope angle b. Therefore, this phenomenon conforms to the principle of infiltration.

**Figure 6:**
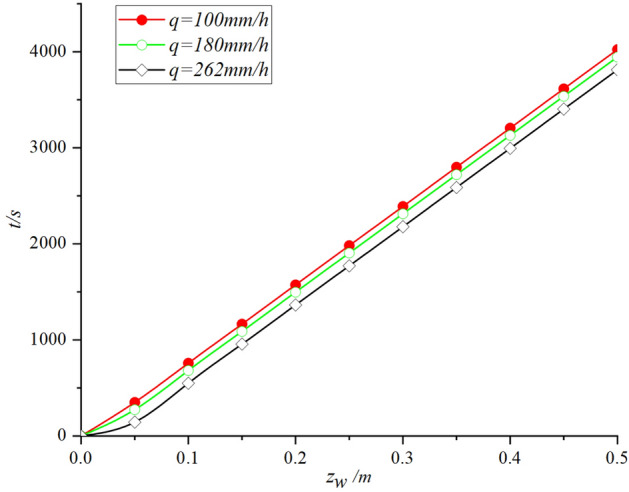
Effect of rainfall intensity on infiltration time.

**Figure 7:**
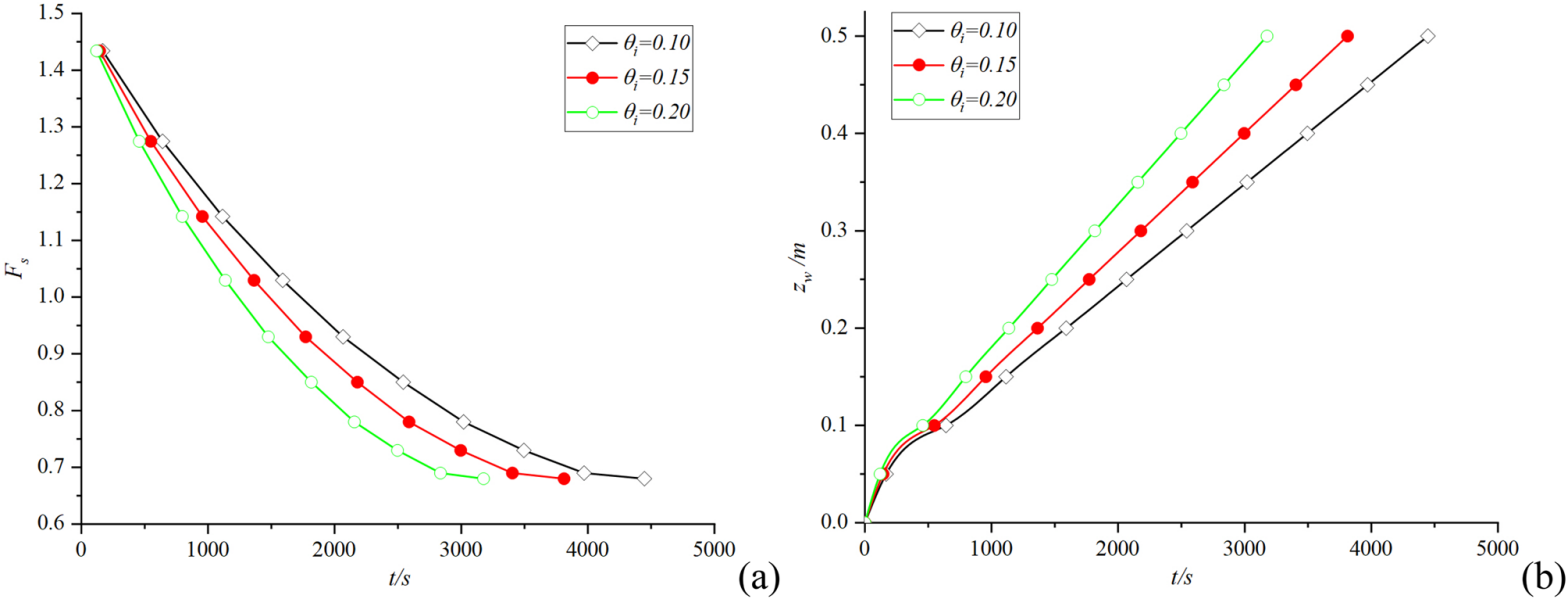
Effect of moisture content on infiltration time.

The influence of rainfall intensity *q* on infiltration time *t* during the whole infiltration process is shown in Fig. 6:

It can be seen from Fig. 6 that the infiltration time *t* decreases with the increase of rainfall intensity *q*, but the difference is not significant. The reason is that because the critical time *t*_*p*_ is very short, the change of wetting front depth *z*_*w*_ with time *t* in the whole infiltration process is less affected by rainfall intensity *q*, which is mainly controlled by hydraulic gradient. Although the erosion of heavy rainfall usually reduces the strength of soil itself and increases the probability of landslide, it has little effect only from the perspective of infiltration theory. The effect of soil initial moisture content *θ*_*i*_ during the whole infiltration process is shown in Fig. 7:

Analysis of Fig. 7a, the higher initial moisture content *θ*_*i*_ of the soil, the faster the safety factor *F*_*s*_ decreases during rainfall infiltration. Figure 7b reveals that the infiltration time *t* is affected by the initial soil moisture content *θ*_*i*_, and the infiltration rate increases with the increase of initial soil moisture content *θ*. It is worth noting that the infiltration depth *z*_*w*_ is not positively correlated with the infiltration time *t* in the period of 0 ~ 500s, because the infiltration depth *z*_*w*_ is mainly controlled by the rainfall intensity *q* before reaching the critical time *t*_*p*_. In summary, we found that when analyzing the stability of soil slopes with high initial moisture content, the redistribution of rainwater in the soil cannot be ignored.

**Figure 8:**
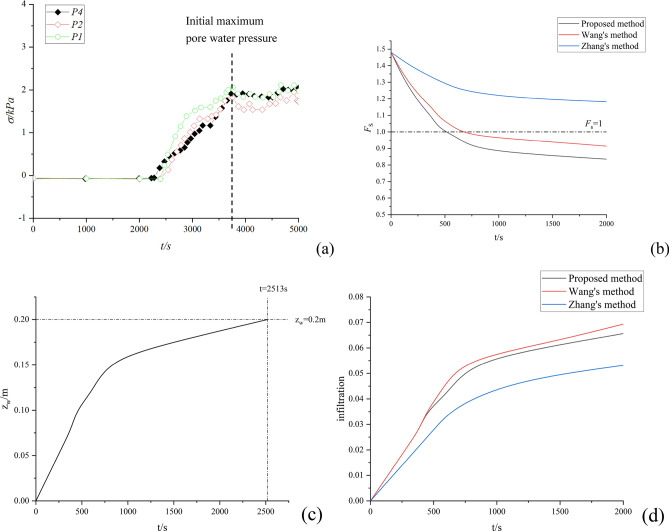
Model verification: (**a**) Experimental monitoring data; (**b**) Comparison of safety factors of different methods; (**c**) The relationship between depth of infiltration and time for the proposed method; (**d**) Relationship between infiltration and time for different methods.

Finally, the calculation results are compared with the experimental monitoring data and other methods to verify the reliability and superiority of the proposed method. As shown in Fig. 8:

As shown in Fig. 8a, the pore water pressure at the 3 monitoring points did not change significantly in the early stage, and started to increase suddenly from about 2250s. This means that water pressure is monitored at infiltration depths up to 0.2 m. Combined with Fig. 8c, this paper's model shows that the infiltration time is 2513 s when the infiltration depth reaches 0.2m. This is in better agreement with the experimental results and shows that the method of this paper is reliable. As shown in Fig. 8b, the method proposed in this paper integrates lateral seepage and air resistance, and *F*_*s*_ decreases gradually with the increase of rainfall duration. Whereas Wang's method only considered lateral seepage, Zhang's method only considered air resistance. Therefore, for the same rainfall duration, the *F*_*s*_ obtained by the method of this paper is smaller compared to the methods of both. This represents the fact that both air resistance and lateral seepage contribute to the reduction of the slope safety factor, so the method in this paper is more relevant to the actual situation. In this case, the air resistance is in the opposite direction of gravity, which reduces the overall slip resistance of the soil. In turn, the penetration of the slope surface increases the overall sliding force on the slope. In addition, the model in this paper calculates the resulting *F*_*s*_ < 1 at 1800s. And the results of the model test showed that the cracks appeared at the top of the slope when the rainfall lasted until about 1800s. This indicates that now the slope has a factor of safety < 1, which is consistent with the calculations in this paper. It also shows the reliability of the method of this paper. As shown in Fig. 8d, air resistance slows the rate of infiltration. This is because during rainfall infiltration, the pore gas pressure increases due to air entrainment. Overall, the method in this paper is more in line with the actual experimental results after incorporating the air resistance hysteresis effect. Thus the advantages of the more reliable and accurate method of this paper are validated.

## Discussion

It is necessary to apply the improved Green-Ampt infiltration model proposed in this paper to finite slope stability test and finite element numerical simulation of simulated rainfall. Because of the different emphasis, some concepts are simplified in the calculation process.

1. Considering the integrity of the slope, the soil blocks are only artificially segmented for the convenience of calculation, so the force between adjacent blocks is not considered in the analysis process.
2. This paper assumes that the matric suction head is constant. However, in fact, some studies have shown that, the water distribution of soil cover is affected by groundwater, and the matric suction head gradually decreases from top to bottom^22^.
3. The gas resistance considered in this paper lacks experimental validation, only by comparing the infiltration time with other tests. Therefore, there are still questions to be addressed in this paper. This paper considered the effect of air resistance on GA models based on Wang's method. The time calculated by Wang's method is 2223 s when the infiltration depth reaches 0.2 m. However, several studies^35,36^ have shown that air resistance can reduce the infiltration rate. Therefore, the infiltration time calculated by the method of this paper is 2513 s. This follows the inference logic. However, the calculations of Wang et al. are closer to the experimental results (2200 s). This leads to a discrepancy between the inference logic and the experimental results. However, due to limitations of our laboratory, we cannot currently conduct large-scale experiments for validation. Therefore, we will conduct an experimental study in future research.

## Conclusion

In this paper, an improved Green-Ampt infiltration model is proposed, which adds the gas lag effect to make it more accurate in analyzing rainfall infiltration of large porosity soils such as sand and laterite. In addition, the relationship between slope size and infiltration process is obtained by combining lateral seepage effect with Darcy's law. In general, this paper lays a preliminary theoretical foundation for the application of large porosity soil slope in finite element numerical simulation. The new method draws the following specific conclusions:

1. The decrease of safety factor slows down with the increase of size, and decreases with the rise of slope angle and initial soil moisture content. The infiltration time increases with the rise of size and slope angle, and decreases with the rise of initial soil moisture content, but is less affected by rainfall intensity.
2. Considering the air resistance hysteresis effect and the influence of lateral seepage, the safety factor reaches the critical value earlier, coinciding with the initial maximum value of pore water pressure.

The proposed method is applicable to finite slopes. On this basis, it is helpful to extend the Green-Ampt infiltration model to two-dimensional and three-dimensional slope models in the future.

## Data Availability

All data in this article can be obtained by contacting the corresponding author.
